# Genome characterization of two novel deep-sea sediment fungi, *Penicillium pacificagyrus* sp. nov. and *Penicillium pacificasedimenti* sp. nov., from South Pacific Gyre subseafloor sediments, highlights survivability

**DOI:** 10.1186/s12864-023-09320-6

**Published:** 2023-05-10

**Authors:** Morgan S. Sobol, Tatsuhiko Hoshino, Victor Delgado, Taiki Futagami, Chihiro Kadooka, Fumio Inagaki, Brandi Kiel Reese

**Affiliations:** 1 Institute for Biological Interfaces 5, Karlsruhe Institute of Technology, Eggenstein-Leopoldshafen, Baden-Württemberg, Germany; 2 Kochi Institute for Core Sample Research, Japan Agency for Marine-Earth Science and Technology (JAMSTEC), Nankoku, Kochi 783-8502 Japan; 3 Department of Life Sciences, TX A&M University, Corpus Christi, Texas USA; 4 Education and Research Center for Fermentation Studies, Faculty of Agriculture, Kagoshima University, Kagoshima, Japan; 5 United Graduate School of Agricultural Sciences, Kagoshima University, 1-21-24 Korimoto, Kagoshima, 890-0065 Japan; 6 Department of Biotechnology and Life Science, Faculty of Biotechnology and Life Science, Sojo University, Ikeda, Nishiku, Kumamoto 860-0082 Japan; 7 Mantle Drilling Promotion Office, Institute for Marine Earth Exploration and Engineering (MarE3), Japan Agency for Marine-Earth Science and Technology (JAMSTEC), Yokohama, 236- 0001 Japan; 8 Department of Earth Sciences, Graduate School of Science, Tohoku University, Sendai, 980-8574 Japan; 9 Dauphin Island Sea Lab, Dauphin Island, Alabama, USA; 10 Stokes School of Marine and Environmental Sciences, University of South Alabama, Mobile, AL USA

**Keywords:** Fungal genome, Marine subsurface, South Pacific Gyre, IODP, Sediment

## Abstract

**Background:**

Marine deep subsurface sediments were once thought to be devoid of eukaryotic life, but advances in molecular technology have unlocked the presence and activity of well-known closely related terrestrial and marine fungi. Commonly detected fungi in deep marine sediment environments includes *Penicillium*, *Aspergillus*, *Cladosporium*, *Fusarium*, and *Schizophyllum*, which could have important implications in carbon and nitrogen cycling in this isolated environment. In order to determine the diversity and unknown metabolic capabilities of fungi in deep-sea sediments, their genomes need to be fully analyzed. In this study, two *Penicillium* species were isolated from South Pacific Gyre sediment enrichments during Integrated Ocean Drilling Program Expedition 329. The inner gyre has very limited productivity, organic carbon, and nutrients.

**Results:**

Here, we present high-quality genomes of two proposed novel *Penicillium* species using Illumina HiSeq and PacBio sequencing technologies. Single-copy homologues within the genomes were compared to other closely related genomes using OrthoMCL and maximum-likelihood estimation, which showed that these genomes were novel species within the genus *Penicillium*. We propose to name isolate SPG-F1 as *Penicillium pacificasedimenti* sp. nov. and SPG-F15 as *Penicillium pacificagyrus* sp. nov. The resulting genome sizes were 32.6 Mbp and 36.4 Mbp, respectively, and both genomes were greater than 98% complete as determined by the presence of complete single-copy orthologs. The transposable elements for each genome were 4.87% for *P*. *pacificasedimenti* and 10.68% for *P*. *pacificagyrus*. A total of 12,271 genes were predicted in the *P*. *pacificasedimenti* genome and 12,568 genes in *P*. *pacificagyrus*. Both isolates contained genes known to be involved in the degradation of recalcitrant carbon, amino acids, and lignin-derived carbon.

**Conclusions:**

Our results provide the first constructed genomes of novel *Penicillium* isolates from deep marine sediments, which will be useful for future studies of marine subsurface fungal diversity and function. Furthermore, these genomes shed light on the potential impact fungi in marine sediments and the subseafloor could have on global carbon and nitrogen biogeochemical cycles and how they may be persisting in the most energy-limited sedimentary biosphere.

**Supplementary Information:**

The online version contains supplementary material available at 10.1186/s12864-023-09320-6.

## Background

Recent surveys of microbial life in the marine subsurface have discovered fungi as a common community member alongside prokaryotes, thanks to advancements in high-throughput sequencing and culturing techniques [1–5]. However, these studies also highlighted how little is known about fungal diversity and their function in deep sea sediments. Largely inaccessible sediments, low biological activity, and difficulty in culturing, are a few of the many challenges scientists face when studying fungi in the deep marine biosphere. So far, marine subsurface environments where fungi have been studied includes: Canterbury Basin [4, 6, 7], Peru Margin and Trench [1–3, 7, 8], Yap Trench [9], North Pond [2], Eastern Equatorial Pacific [2], Hydrate Ridge [2], Magellan Seamounts [10], Benguella Upwelling System [2], various sites within the Indian Ocean [11–14], and Japan’s Suruga-Bay [15] and Shimokita Peninsula [5], and even within South Shetlands Islands, Antarctica [16]. The studies above used culture dependent and culture independent methods to assess the fungal diversity but only a few [3, 4, 7–9, 14, 16] were able to make conclusions on the activity and subsequently, the role of fungi in deep sea sediments leaving much to still learn about fungi in this environment.

In terrestrial environments, fungi are notoriously saprotrophic, meaning they actively breakdown recalcitrant non-bioavailable organic matter (e.g., polycyclic aromatic hydrocarbon, lignin, lignocellulose, carboxylic acids) subsequently turning it into labile carbon [17–20]. Recent studies have found that many marine subsurface fungi are closely related to well-known saprotrophic fungi such as *Aspergillus*, *Penicillium*, *Fusarium*, *Cryptococcus*, and *Schizophyllum* [1, 2, 4, 5, 14, 21].

**Fig. 1:**
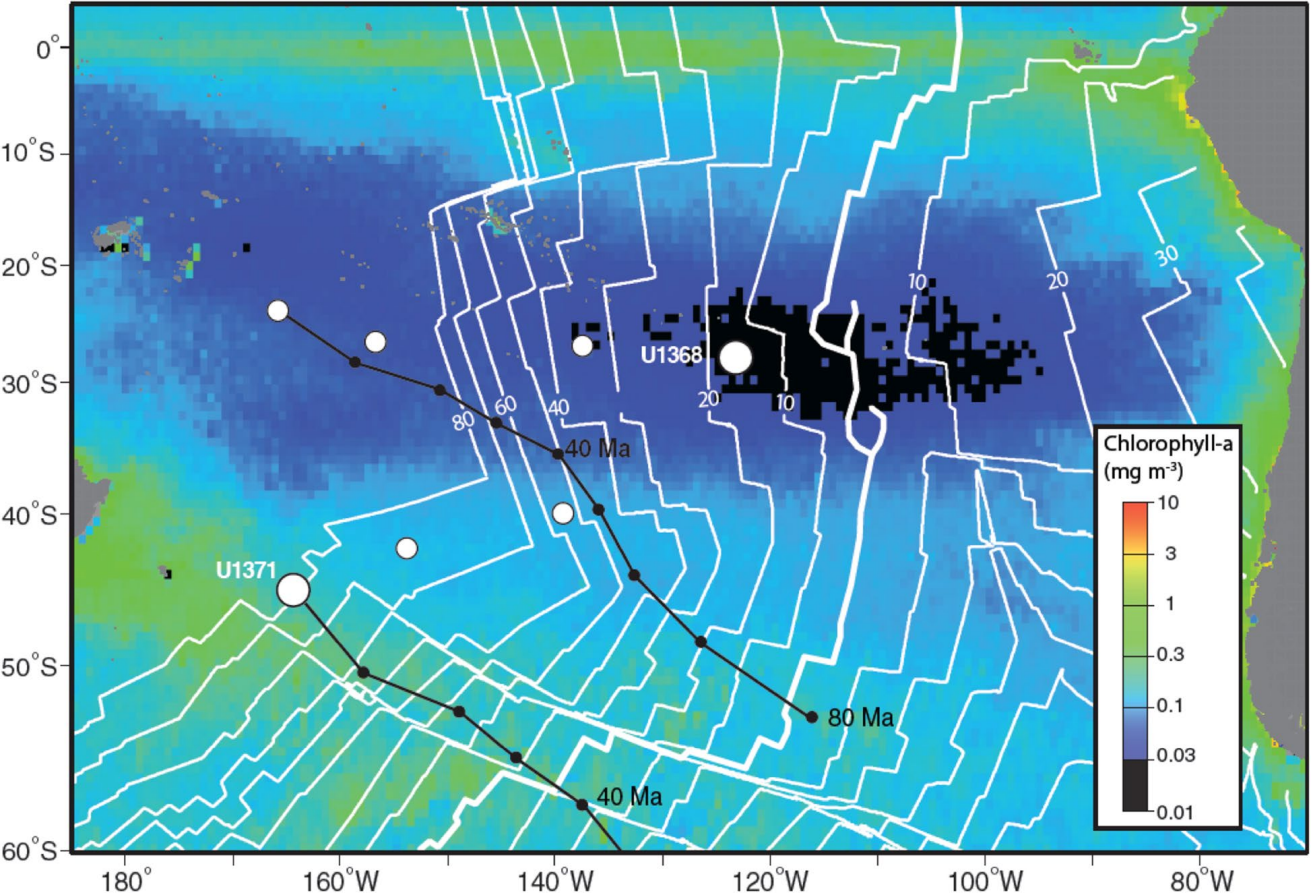
Location of Holes U1371 and U1368 overlaying annual chlorophyll-a concentrations. Whole round cores were taken from these sites during IODP Expedition 329 to the South Pacific Gyre from October – December 2011. White lines indicate basement age in 10 mya increments. Black lines indicate site positions through geologic time. The figure was modified from [25]

Fungi in marine habitats are important members of the carbon cycle by remineralizing recalcitrant carbon, supplying energy-limited sediment with labile carbon through their necromass, and the provision of water and nutrients [22, 23]. Invariably, marine sedimentary fungi have been overlooked in recent global biomass estimates [1, 24]. Here, we interrogated the genomes of two marine subsurface fungi isolated from South Pacific Gyre (SPG) sediment enrichments for genes related to recalcitrant carbon and nitrogen degradation and survival in oligotrophic sediments. Sediments from the SPG have been described as some of the most energy-limited on Earth, with cell abundances 3 to 4 orders of magnitude less than other deep-sea sediments [25]. Sediment was collected via the Integrated Ocean Drilling Program (IODP) Expedition 329 to the SPG (Fig. 1) from two locations and two depths (U1371E-14H2 at 124 m below seafloor (mbsf) and U1368D-2H1 at 12 mbsf). We used both genomes to evaluate the phylogenomic placement alongside reference *Penicillium* and *Aspergillus* genomes and determined that both isolates were new species within the *Penicillium* genus. Both genomes contained genes associated with the degradation of aromatic compounds, lignin, lignocellulose, carbonate, and carboxylic acids. Genes related to stress and nutrient response, DNA repair, and secondary metabolite synthesis were also explored in both genomes. With the presence of these genes, we present evidence on the metabolic capabilities of both SPG fungal isolates and their survivability in deeply buried SPG sediments for millions of years. This study provides an important first characterization of whole genomes from subseafloor sediment *Penicillium* isolates.

## Results

### Genome statistics

Illumina Hiseq sequencing of the genomes resulted in 149,610,352 reads (15,110,645,552 bp) for *Penicillium* sp. SPG-F1 (from IODP hole U1371E-14H2) and 162,517,156 reads (16,414,232,756 bp) for *Penicillium* sp. SPG-F15 (from IODP hole U1368D-2H1). Assuming an average genome length of 32 Mega base pairs (Mbp) for *Penicillium* [26], we estimated the sequencing coverage depth to be 513X for *Penicillium* sp. SPG-F15 and 472X for *Penicillium* sp. SPG-F1.

PacBio sequencing resulted in 484,101 subreads (4.45 Giga base pairs, Gbp) for *Penicillium* sp. SPG-F1 and 472,422 subreads (4.24 Gbp) for *Penicillium* sp. SPG-F15. The average read length of the reads for *Penicillium* sp. SPG-F1 was 9,186 bp and 8,978 bp for *Penicillium* sp. SPG-F15. The total coverage using PacBio was 132X for *Penicillium* sp. SPG-F15 and 139X for *Penicillium* sp. SPG-F1. A total of 2.2% of all base pairs was trimmed from *Penicillium* sp. SPG-F15 and 3.0% of bp was trimmed from SPG-F1.

By combining both PacBio and Illumina reads, we assembled two genomes de novo (i.e., without a reference) (Table 1). The hybrid assembly for *Penicillium* sp. SPG-F1 was 32.6 Mbp in length and resulted in 62 contigs greater than 500 bp, the N50 of the assembly was 1,348,856 bp, and the GC content was 48.23% (Table 1). The hybrid assembly for *Penicillium* sp. SPG-F15 was 36.4 Mbp in length with 48 contigs greater than 500 bp, N50 of 3,205,559 bp, and the GC content was 46.46%. Our assembled genomes fell within the size range of other *Penicillium* species, which is typically between 24 and 36 Mbp [26]. The completeness of *Penicillium* sp. SPG-F1’s assembly, according to BUSCO [27], included 748/758 complete marker genes (98.7%) (Table 1). The assembly for *Penicillium* sp. SPG-F15 contained 749/758 (98.8%) (Table 1). Contamination, estimated as the percentage of duplicate fungal marker genes, was found to be < 1.0% for both isolates using BUSCO [27] (Table 1). Additionally, NCBI found no contamination from other organisms upon submission.

**Table 1:** Genome statistics for *P*. *pacificasedimenti* sp. nov. and *P*. *pacificagyrus* sp. nov

	***P*. *pacificasedimenti***	***P*. *pacificagyrus***
***Assembly Features***		
N50 (bp)	1,348,856	3,205,559
L50	7	3
No. of Contigs (> 500 bp)	62	48
Largest Contig (bp)	3,064,773	10,386,503
Genome Size (Mbp)	32.6	36.4
GC Content	48.23%	46.46%
***Masked Repeats***		
Total TEs	4.13%	9.86%
SINEs	0.00%	0.00%
LINEs	0.38%	0.20%
LTR Elements	0.66%	4.53%
DNA Elements	2.04%	0.31%
Unclassified	1.05%	4.81%
Simply Repeats	0.62%	0.69%
Low Complexity	0.14%	0.14%
***Annotation Features***		
BUSCO Completeness^a^	98.70%	98.80%
BUSCO Contamination^a^	0.9%	0.5%
Total No. of Genes	12,271	12,568
Total No. of GOs	6,760	6,618

^a^Completeness and contamination estimations were based on BUSCO [27] using universal fungal-specific marker sets. Completeness is estimated from the number of complete marker genes, whereas contamination is estimated based on the number of duplicated marker genes

A total of 3,890,999 bp (10.68%) of repetitive elements were masked in *Penicillium* sp. SPG-F15; whereas, *Penicillium* sp. SPG-F1 had 1,590,760 bp (4.87%) that were masked (Table 1). Transposable elements (TEs) made up most of the repeats for both isolates (*Penicillium* sp. SPG-F15 9.86%, *Penicillium* sp. SPG-F1 4.13%). Approximately, 50% of TEs in *Penicillium* sp. SPG-F15 and 25% of TEs in *Penicillium* sp. SPG-F1 were unclassified. The rest of the repetitive elements were classified as simple repeats and low complexity repeats. Simple repeats made up 0.62% of the genome in *Penicillium* sp. SPG-F1 and 0.69% in *Penicillium* sp. SPG-F15. Low complexity repeats comprised of 0.14% of the genome in both isolates.

**Fig. 2:**
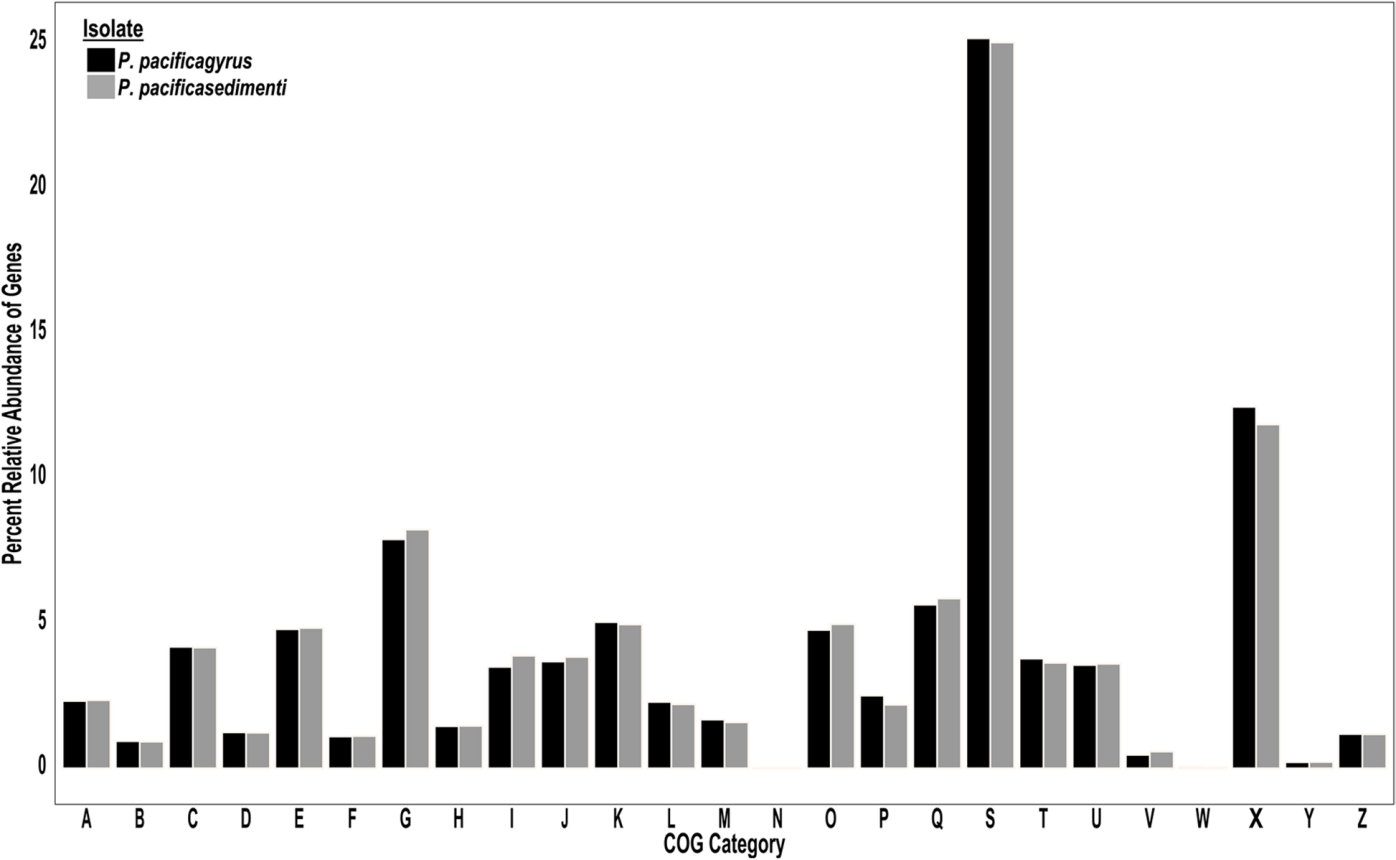
Functional annotation with COG categories: [A] RNA processing and modification, [B] Chromatin structure and dynamics, [C] Energy production and conversion, [D] Cell cycle control, cell division, chromosome partitioning, [E] Amino acid transport and metabolism, [F] Nucleotide transport and metabolism, [G] Carbohydrate transport and metabolism, [H] Coenzyme transport and metabolism, [I] Lipid transport and metabolism, [J] Translation, ribosomal structure and biogenesis, [K] Transcription, [L] Replication, recombination and repair, [M] Cell wall/membrane/envelope biogenesis, [N] Cell motility, [O] Post-translational modification, protein turnover, and chaperones, [P] Inorganic ion transport and metabolism, [Q] Secondary metabolites biosynthesis, transport, and catabolism, [S] Function unknown, [T] Signal transduction mechanisms, [U] Intracellular trafficking, secretion, and vesicular transport, [V] Defense mechanisms, [W] Extracellular structures, [X] COG not assigned, [Y] Nuclear structure, [Z] Cytoskeleton

MAKER2 predicted a total of 12,568 protein coding genes for *Penicillium* sp. SPG-F15 and 12,271 protein coding genes for *Penicillium* sp. SPG-F1 (Table 1). Of these genes, a total of 11,396 COG/KOG categories were assigned to 11,033 genes annotated with eggNOG-mapper v.4.5.1 [28, 29] for *Penicillium* sp. SPG-F1. As for *Penicillium* sp. SPG-F15, 11,828 COG/KOG’s out of 11,458 genes annotated were assigned to *Penicillium* sp. SPG-F15. The top three COG/KOG categories for both isolates, in order, were “Function Unknown” [S], “No COG/KOG Assigned” [X], and “Carbohydrate Transport and Metabolism” [G], which represented 25%, 12%, and 8% of the total COG/KOG assignments, respectively (Fig. 2). Approximately 35% of each genome could not be annotated via eggNOG-mapper v.4.5.1 [28, 29], and approximately 5% of the total COG/KOG annotations were identified as hypothetical proteins.

**Fig. 3:**
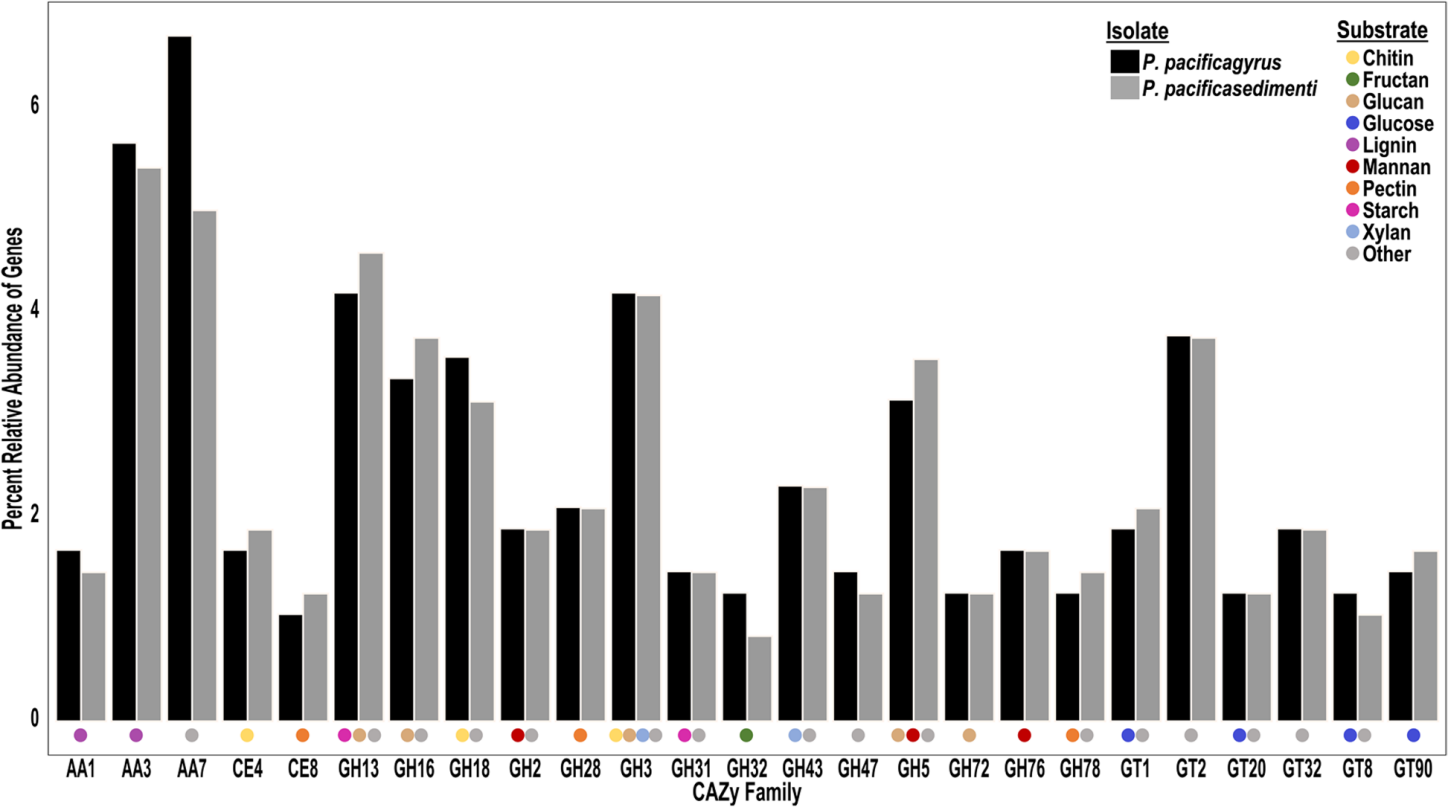
Functional annotation of the most abundant CAZy annotations and their substrates. Only the families representing a relative abundance of 1.25% or greater were included here and include AA: auxiliary activities, CE: carbohydrate esterases, GH: glycoside hydrolases, GT: glycosyl transferases. Substrates listed as “Other” can be found in more detail in Additional file 1, Supplementary Table 2. [AA1] Multicopper oxidases/laccases, [AA3] Glucose-methanol-choline oxidoreductases, [AA7] Glucooligosaccharide oxidases, [CE4] Acetyl xylan esterases, chitin/peptidoglycan deacetylases, [CE8] Pectin methylesterases, [GH13] α-1,3-glucan synthases, [GH16] endo-1,3(4)-β-glucanases, [GH18] Chitinases, [GH2] β-galactosidases, β-mannosidases, β-glucuronidases, [GH28] polygalacturonases, α-L-rhamnosidases, [GH3] β-glucosidase, [GH31] α-glucosidases, α-galactosidases, [GH32] endo-inulinases, fructan β-fructosidases, [GH43] β-xylosidase, α-L-arabinofuranosidase, [GH47] α-mannosidase, [GH5] endo-β-1,4-glucanase/cellulase, [GH72] β-1,3-glucanosyltransglycosylase, [GH76] α-1,6-mannanase, α-glucosidase, [GH78] α-L-rhamnosidase, rhamnogalacturonan α-L-rhamnohydrolase, [GT1] UDP-glucuronosyltransferase, [GT2] cellulose synthase, chitin synthase, [GT20] α,α-trehalose-phosphate synthase, [GT32] α-1,6-mannosyltransferase, [GT8] lipopolysaccharide α-1,3-galactosyltransferase, [GT90] glucuronoxylomannan/galactoxylomannan β-1,2-xylosyltransferase

A total of 480 genes from *Penicillium* sp. SPG-F1 (3.9% of the total) and 477 genes from *Penicillium* sp. SPG-F15 (3.8% of the total) encoded for carbohydrate-active enzymes, according to the CAZy database (Fig. 3). The enzymes within the CAZy database were divided into classes and families, and assigned a numerical value (e.g., AA1). In *Penicillium* sp. SPG-F15, a total of 60 proteins encoded for carbohydrate-binding modules (CBM), 116 proteins encoded for carbohydrate esterases (CE), 253 proteins encoded for glycoside hydrolases (GH), 97 proteins encoded for glycosyl transferase (GT), 9 proteins encoded for polysaccharide lyases (PL), and 91 proteins encoded for auxiliary activities (AA) (Fig. 3). In SPG-F1, a total of 62 proteins encoded for CBM, 119 proteins encoded for CE, 270 proteins encoded for GH, 104 proteins encoded for GT, 12 proteins encoded for PL, and 86 proteins encoded for AA. No single family represented more than 7% of all CAZy annotations for each genome (Fig. 3).

**Fig. 4:**
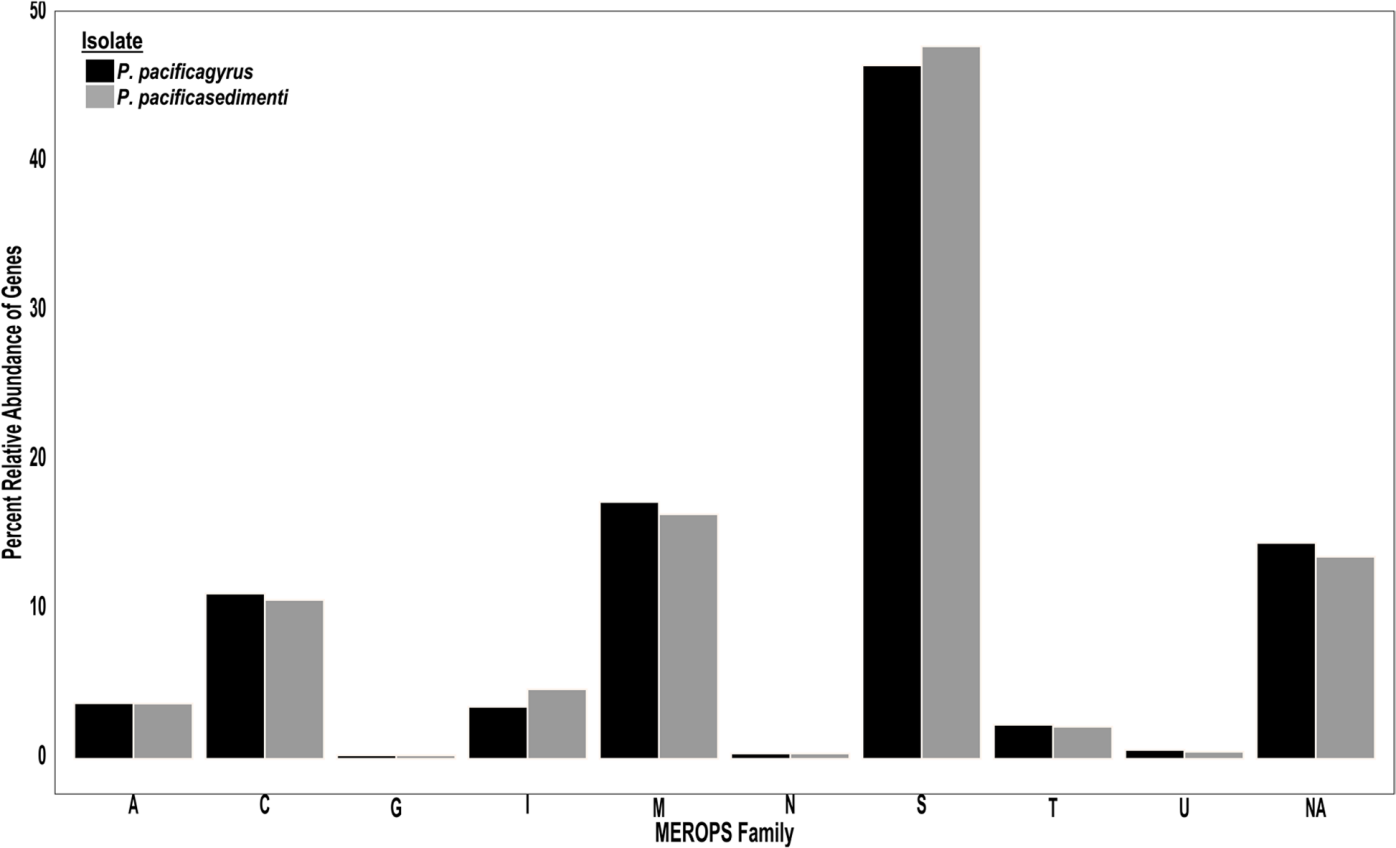
Functional annotation of protease families using the MEROPS database (Rawlings et al. 2018). [A] Aspartic, [C] Cysteine, [G] Glutamic, [I] Inhibitor, [M] Metallo, [N] Asparagine, [S] Serine, [T] Threonine, [U] Unknown, [NA] Not Assigned

Annotation with the MEROPS database resulted in a total of 835 genes annotated (6.8% of the total) for *Penicillium* sp. SPG-F1 and 830 genes annotated (6.6%) for *Penicillium* sp. SPG-F15 (Rawlings et al. 2018). The most abundantly found peptidases were the serine peptidases, totaling almost 50% of all MEROPS annotations (Fig. 4). Genes that were assigned a function, but not to a particular family were listed as NA and accounted for approximately 14% of genes in both fungi (Fig. 4).

A search of the KEGG Automatic Annotation Server (KAAS) database [30, 31] resulted in 3,811 (31% of total genes) KEGG orthologies (KO) assigned for *Penicillium* sp. SPG-F1 and 3,847 (31% of total genes) KOs for *Penicillium* sp. SPG-F15. Most KOs were assigned to metabolic pathways (ko01100), biosynthesis of secondary metabolites (ko01110), and biosynthesis of antibiotics (ko01130) for both isolates. All functional annotations were further analyzed for specific genes involved in carbon and nitrogen linked metabolisms, cellular maintenance, and stress responses.

### Phylogenomics

**Fig. 5:**
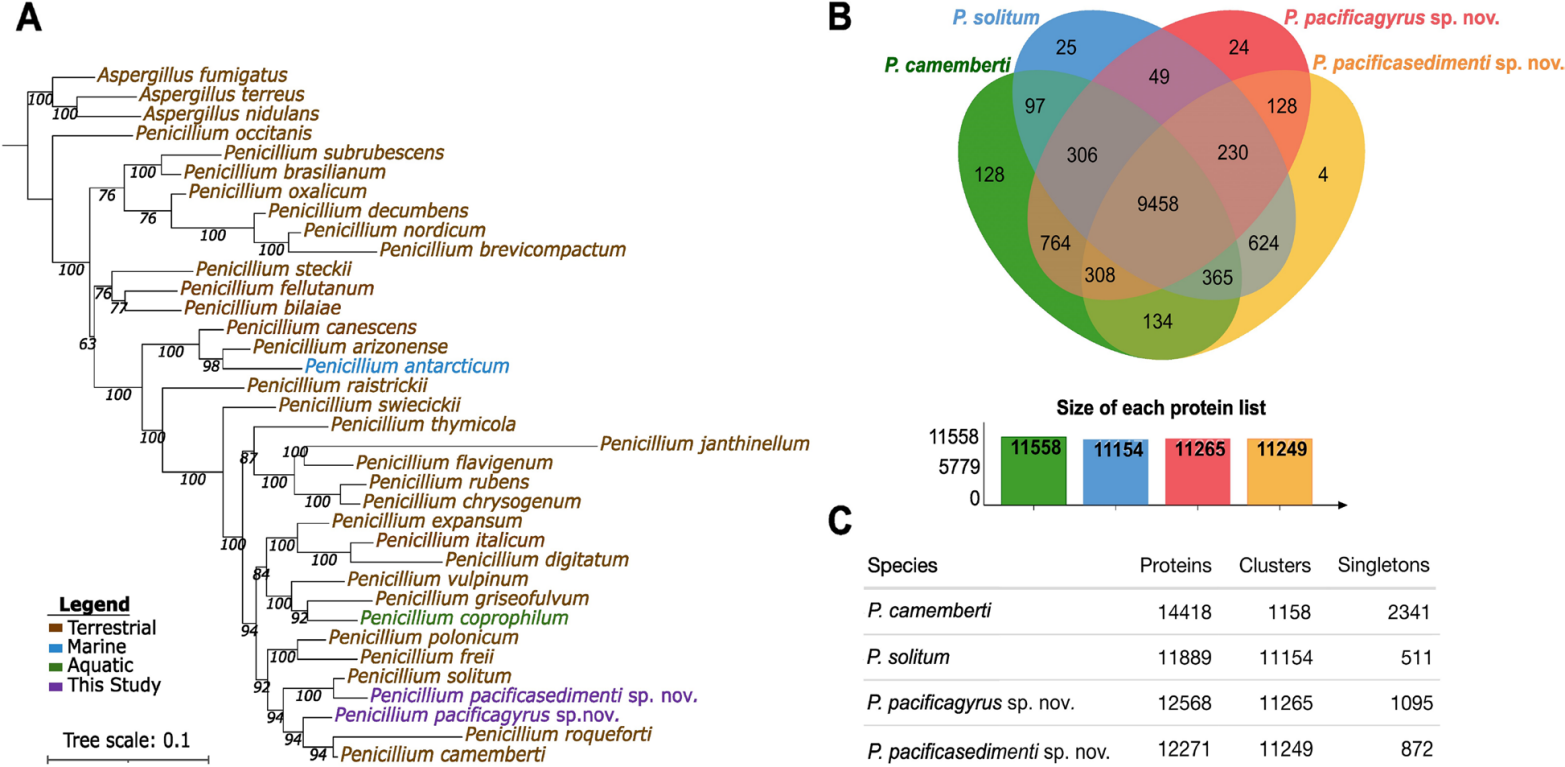
Pangenomic analysis. **A** Whole genome phylogenetic analysis of *P*. *pacificasedimenti* and *P*. *pacificagyrus* conducted using concatenated single-copy marker genes determined by GET_HOMOLOGUES [36] which used the OrthoMCL algorithm. The tree was estimated using maximum likelihood analysis and 1,000 bootstraps in IQ-TREE [37]. Bootstrap support values are shown at each node. The best-fit model chosen by ModelFinder [38] was GTR2 + FO + ASC + G4. The tree was re-rooted with *Aspergillus* species as the outgroup and visualized with iTOL [39]. **B** Venn diagram generated with OrthoVenn2 [40] showing the number of shared and unique protein clusters between our isolates and their closest relative. **C** Summary of the number of proteins, protein clusters, and single proteins within each genome

Phylogenomics was used to understand the whole genome placement of both *Penicillium* sp. SPG-F15 and *Penicillium* sp. SPG-F1 within the *Penicillium* clade (Fig. 5a) as our previous identification using 18S rRNA [32] and ITS (data not shown) did not have sufficient resolution for precise identification, which is a known issue for *Penicillium* [33]. Average Amino acid Identity (AAI) was then compared between both isolates and their closest relative based on Fig. 5. *Penicillium* sp. SPG-F1 shared a 97.78% similarity with *Penicillium solitum* and *Penicillium* sp. SPG-F15 shared a 97.24% similarity with *Penicillium camemberti*. Based on recent proposed species cut-off of < 99% for fungi [34, 35], we propose the new names *Penicillium pacificasedimenti* sp. nov. for *Penicillium* sp. SPG-F1 and *Penicillium pacificagyrus* sp. nov. for *Penicillium* sp. SPG-F15.

Further comparison of putative orthologous protein clusters between *P*. *pacificasedimenti, P*. *pacificagyrus*, *P*. *camemberti*, and *P*. *solitum* revealed that all species shared 9458 clusters (Fig. 5b). *P*. *pacificasedimenti* and *P*. *solitum* shared 624 clusters and *P*. *pacificagyrus* and *P*. *camemberti* shared 764. Our two isolates shared 128 clusters. *P*. *pacificasedimenti* had 4 unique clusters with 3 clusters having an unknown GO annotation and 1 related to GO:0030245; cellulose catabolic processes. *P*. *pacificagyrus* had 24 unique clusters of which 17 have no annotation, whereas the other 7 clusters are related to melanin biosynthetic process (GO:0042438), metal ion transport and response (GO:0030001, GO:0010038), response to arsenic-containing substances (GO:0046685) and regulation of cyclin-dependent protein serine/threonine kinase activity (GO:0000079). *P*. *pacificasedimenti* and *P*. *pacificagyrus* had 872 and 1,095 proteins, respectively, that were not in any cluster (Fig. 5c).

### Cell maintenance and stress response

Annotations from the COG/KOG and KAAS analysis indicated many genes related to the maintenance and survival of the fungal cell in our *Penicillium* genomes such as DNA repair, cell wall maintenance, and autophagy related genes. Genes from the Radiation (Rad) family involved in DNA repair (i.e., Rad-1, Rad-4, Rad-7, Rad-10, Rad-14, Rad-16, Rad-18, Rad-26, Rad-51, Rad-52, Rad-57) were located in both genomes. Also included were genes in the mismatch repair (MMR) family such as MutL homolog 1 (MHL1), MLH3, MutS homolog 1 (MSH1), and MSH2. Other maintenance genes found included minichromosome maintenance (MCM4), methyl methanesulfonate 21 (MMS21), methyl methansulfonate, UV sensitive 81 (MUS81), and psoralen 2 (PSO2). There were many genes associated with the AuTophaGy (ATG) gene family within both genomes, which is important for cellular homeostasis. These included ATG1, ATG2, ATG3, ATG4, ATG5, ATG6, ATG7, ATG8, ATG9, ATG11, ATG12, ATG13, ATG15, ATG17, ATG18, ATG20, ATG22, ATG24, ATG26, and ATG27. Genes involved in the ubiquitin–proteasome degradation pathway, which were found in both of our genomes, included ubiquitin ligases (K10592, K10625, K10590, K14023, K14026, K03347), ubiquitin hydrolases (K05609, K05610, K11836, K11838, K11839, K11849, K11366), and the components making up the 20S/26S proteosome complexes (K03028, K03029, K06693, K03032, K11599, K03350, K03353, K03348, K03355). Genes associated with cell wall maintenance (i.e., CAZy GH13 α-glucan synthase) were identified in *P*. *pacificagyrus* (20 copies) and in *P*. *pacificasedimenti* (22 copies). Also found were 18 copies of GT2 (cellulose/chitin synthase) in both genomes (Fig. 3).

### Peptide metabolism

**Fig. 6:**
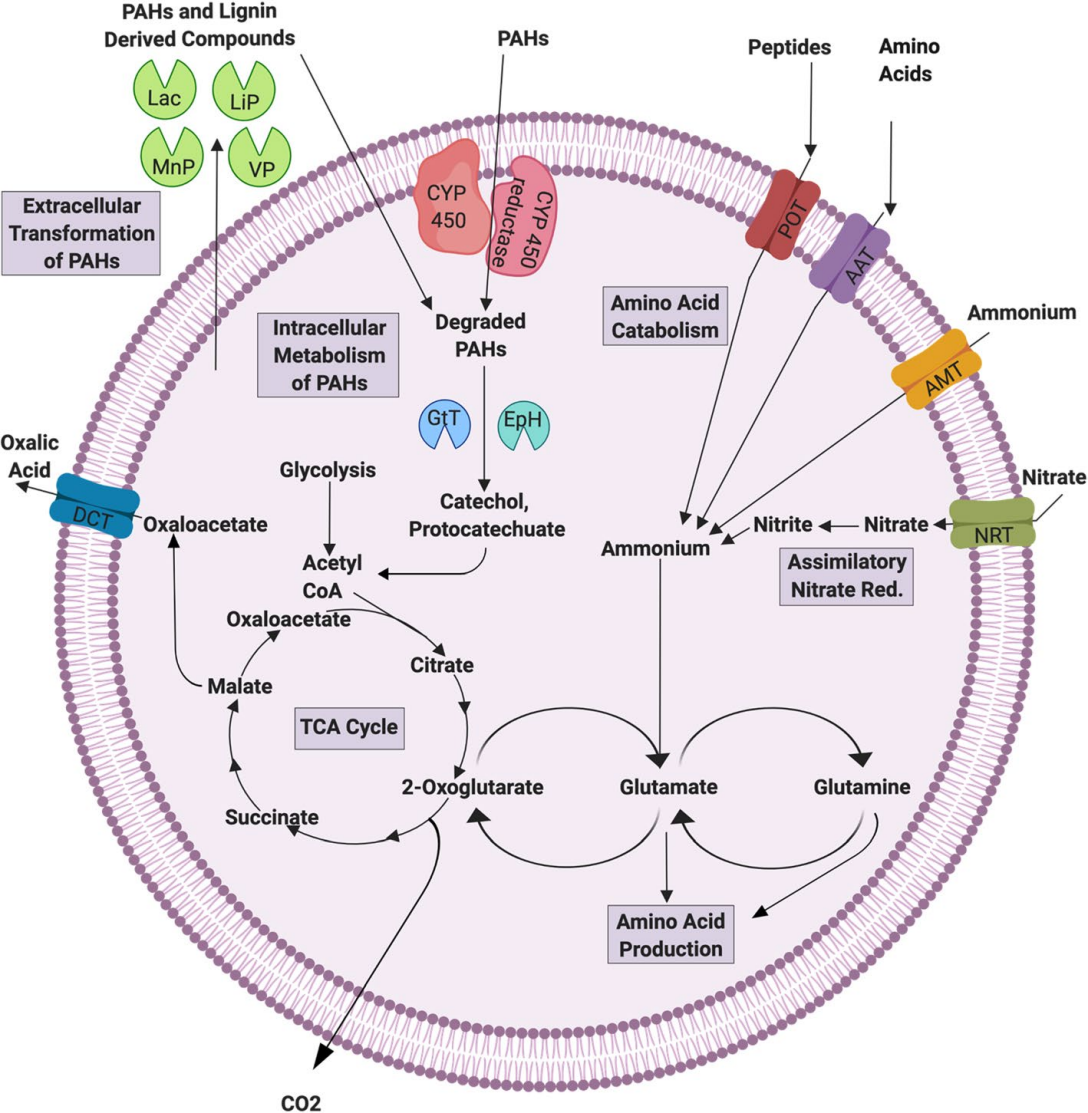
Cellular metabolic model for *P*. *pacificasedimenti* sp. nov. and *P*. *pacificagyrus* sp. nov., focusing on carbon and nitrogen metabolisms. Created with BioRender.com

The fungi were examined for the ability to metabolize extracellular amino acids, peptides, and other proteinaceous material via the KAAS, MEROPS, and COG annotations (Fig. 6). Both fungi had one amino acid transporter (AAT) K03293, one proton-coupled AAT K14209, one sodium-coupled neutral AAT K14997, and 23 yeast AAT K16261. Isolate *P*. *pacificasedimenti* also had 19 AAT that were not assigned a KO number, *P*. *pacificagyrus* had 21 not assigned a KO. A total of 17 oligopeptide transporters (OPT) were found in *P*. *pacificagyrus* and 15 in *P*. *pacificasedimenti* (no KEGG KO given). Four copies of the proton-dependent oligopeptide transporter (POT; K03305) were identified in both genomes. The two isolates also contained genes required to secrete extracellular peptidases such as aminopeptidase (one K05994, one K01268, one K13721, one K01267), carboxypeptidase (seven K01288, two K13289, one K08783, two K01301, two K01293), one dipeptidyl-peptidase (K01282), two tripeptidyl-peptidase (K01279), and three dipeptidases (K01273).

### Carbohydrate metabolism

Based on the carbon metabolism pathways (ko01200) and the starch and sucrose pathway (ko0050) from the KASS annotation, both of the fungi can use a variety of carbohydrate substrates. These include: methanol, formaldehyde, formamide, glucose, fructose, glyceraldehyde, isomaltose, starch, glycogen, sucrose, cellulose, and trehalose. Enzymes which use glucose, sucrose, fructose, starch, glycogen, and cellulose as substrates were additionally found encoded in both genomes based on CAZy annotations (Fig. 3). In the COG/KOG annotations several sugar transporters involved in the transport of these carbohydrate substrates were identified to further provide evidence for their diverse metabolism. These included lactate transporter (K08178), myo-inositol transporter (K08150), sugar (and other) transporter (K08141 or no KO provided), glucose transporter (no KO provided), sucrose transporter (K15378), and galactose transporter (K15272). Other transporters identified to be involved in carbohydrate metabolism included the major facilitator superfamily (MFS) transporters (K08139, K08141, K08150, or no KO provided).

### Recalcitrant carbon degradation

Genes involved in polycyclic aromatic hydrocarbon (PAH) degradation via cytochrome P450 (CYP 450) monooxygenase enzymes in both fungal isolates. These include CYP 450 monooxygenase (K21293, K22992), benzoate 4-monooxygenase CYP 450 (K07824), and NADPH-cytochrome P450 reductase (K14338) (Fig. 6). According to COG/KOG annotations, there were 119 genes in *P*. *pacificagyrus* classified as CYP 450, and 122 in *P*. *pacificasedimenti*. Both fungal genomes also had genes that encoded enzymes involved in extracellular lignin and PAH degradation determined through CAZy annotation. These enzymes included: laccase (Lac; AA1), lignin peroxidase (LiP, AA2), manganese peroxidase (MnP; AA2), and versatile peroxidase (VP; AA2). CAZy family AA1 comprised 1.5% of all CAZy annotations for *P*. *pacificasedimenti* and 1.7% of *P*. *pacificagyrus*. The relative abundance of family AA2 was 0.6% of all CAZy annotations for both genomes. Epoxide hydrolase (K01253; EpH), and glutathione transferase (K00799; GtT) were two of the intracellular enzymes found in both genomes. A total of six EpH’s were found in *P*. *pacificasedimenti* and eight in *P*. *pacificagyrus*, whereas 22 GtT’s were found in both genomes.

### Nitrogen metabolism

According to KAAS annotations, both fungi did not include genes associated with dissimilatory nitrate reduction, but contained genes involved in nitrate and ammonium assimilation. Specifically, the genes involved in these two pathways found were endopeptidases, ammonium transporter (AMT, K03320), the nitrate transporter (NRT, K02575), nitrate reductase (NR, K10534) and nitrite reductase (NIT-6, K17877) (Fig. 6). Three copies of AMT and one copy of NRT were identified in both genomes. Four NR’s were found in *P*. *pacificagyrus* and two found in* P*. *pacificasedimenti*. One NIT-6 was found in each genome.

### Secondary metabolites

According to the antibiotics and secondary metabolites analysis shell database (antiSMASH) [41], a total of 52 secondary metabolites were found in *P*. *pacificagyrus* and 46 were found in *P*. *pacificasedimenti*. Two of these metabolites, shared between both fungi, had a 100% similarity to napthopyrone which is a Type 1 polyketide synthase (T1PKS) and aureobasidin A1, which is a non-ribosomal polyketide synthase (NRPS). They both also shared a 75% match to nidulin A (NRPS). They also individually had unique matches. Isolate *P*. *pacificasedimenti* had a region with a 100% match to alternariol (T1PKS). *P*. *pacificagyrus* had 100% similarity matches to nectrisine (NRPS), deoxysambucinol (terpene), rhizomide (NRPS), and PR toxin (terpene). This isolate also had a 71% match to cyclopiazone acid made up of NRPS, T1PKS, and indole. A large portion of both isolates had no similar known secondary metabolite match, or metabolites with very low similarity (less than 70%). When combined, these unknowns or low similarity metabolites, made up 85% of the total matches for *P*. *pacificagyrus,* and 89% of the total matches for *P*. *pacificasedimenti*.

Further evidence for putative antimicrobial and secondary metabolite synthesis was investigated from the COG/KOG annotations. In both genomes, 10 genes were classified as nonribosomal peptides and NRPS-like enzymes. One nonribosomal siderophore peptide synthase was also found in each genome. A total of 27 genes matched to polyketide synthase (PKS), and one hybrid NRPS PKS enzyme was found in both genomes. Additionally, numerous MFS and ATP-binding cassette (ABC) transporters potentially involved the transport of secondary metabolites were annotated.

### Description of Penicillium pacificasedimenti sp. nov***.***

#### Etymology

*pacificagyrus* refers to the South Pacific Gyre located in the Pacific Ocean, from where the organism was isolated.

#### Standard description

The fungal colonies were 12 mm in diameter on average after 14 days of growth on potato dextrose agar (PDA) at 5ºC in oxic conditions. Under anoxic conditions, the isolate grew slower than under aerobic conditions. The colonies were sulcate, velutinous, having dense margins, narrow, and slightly floccose. Regarding the color, the periphery was white, the center a dull-green (Munsell color 25-28D-E3) to dark green (Munsell color 28F5), and the reverse was yellow. Moderate conidiogenesis was observed. The isolate also grew under anoxic conditions, but the growth was slower. Microscopically, the conidia were ellipsoidal, hyaline, and 2.8–3.3 µm long. The stipes were 300–500 µm long, hyaline, and typically bearing terverticallite and quaterverticillate penicilli, and sometimes biverticillate. Growth occurred between 4ºC and 30ºC, 1% to 12% NaCl, and within pH 3 to 8. Dissimilatory nitrate reduction and dissimilatory sulfate reduction were both negative. Fermentation and lignin degradation were observed. More detailed growth characteristics can be found in Kiel Reese et al. (2021) where the ecophysiology of the isolates was analyzed in depth [32]. Whole genomic comparison of single-copy marker genes and average amino acid identity phylogenetically placed this isolate as a new species of the genus *Penicillium*.

### Description of Penicillium pacificasedimenti sp. nov.

#### Etymology

*pacificasedimenti* refers to the deep sediment, near the South Pacific Gyre, from where the organism was isolated.

#### Standard description

The fungal colonies were 21 mm in diameter on average after 14 days of growth on potato dextrose agar (PDA) at 5ºC in oxic conditions. Growth was also observed under anoxic conditions, but was slightly slower. Morphologically, the colonies were sulcate, velutinous-flocculent, with a white to yellowish outermost margin and greyish turquoise to dull green colonies (Munsell colors 24-25D3, 26-27E3-4). The reverse was yellow and the exudate a light brown. An abundance of conidiophores was promptly produced after germination, which were microscopically found to be 300–400 µm long. The conidia themselves were ellipsoidal to subspheroidal in shape and 2.9 µm to 3.5 µm. Other microscopic characteristics showed that the penicillin was terverticallite, with few occurrences of quaterverticillate. Growth occurred between 4ºC and 30ºC, 1% to 12% NaCl, and within pH 3 to 8. Dissimilatory nitrate reduction and dissimilatory sulfate reduction were both negative. Fermentation and lignin degradation were observed. More detailed growth characteristics can be found in Kiel Reese et al. (2021) where the ecophysiology of the isolates was analyzed in depth [32]. Whole genomic comparison of single-copy marker genes and average amino acid identity phylogenetically placed this isolate as a new species of the genus *Penicillium*.

## Discussion

### The oligotrophic South Pacific Gyre

The South Pacific Gyre is the furthest site away from productive ocean regions and continents (Fig. 1) than any other place on Earth [25], therefore, organic carbon input from terrestrial environments and surface waters is very low. Some of the lowest recorded sedimentation rates in the global ocean are found at SPG sites. Site U1368, which was located within the gyre itself, had a calculated sedimentation rate of 1.11 m myr ^−1^, in comparison to site U1371, which was located directly outside of the gyre and had a calculated sedimentation rate of 1.78 m myr ^−1^. Based on sedimentation rates, *Penicillium pacificagyrus* was isolated from sediments aged 11 million years before present (mybp) and *Penicillium pacificasedimenti* from sediments aged 70 mybp. At all sites within SPG IODP Expedition 329 (U1365-U1370), total organic carbon (TOC) declined rapidly at the surface and after 1.5 mbsf, TOC remained below 0.05 wt%. TOC in site U1371E located just outside the gyre declined rapidly at the surface as well, but remained at a concentration of 0.1% throughout most of the sediment column until it further declined to ~ 0.05 wt% at 101 mbsf [25]. Also, the CaCO_3_ concentrations within hole U1371E varied from 0.04 wt% at the surface to 0.78 wt% at depth, and 60 wt% at the surface to 87 wt% at depth within hole U1368D [25].

Previous evidence has found fungal like hyphae in deep-sea sediments [7, 42, 43] and potentially active expression of fungal genes [2, 3, 7]. We previously detected 18S rRNA transcripts most closely related to *Penicillium* within the isolate’s sister sediment samples that were cryogenically preserved while on the ship, described herein [32]. However, whether fungi are active or exist as dormant spores in deep-sea sediments deposited via aeolin transport, remains poorly understood. Therefore, it is important to understand the extent of fungi’s metabolic capabilities and their role in biogeochemical cycling within these sediments, but we must also be cautious in how it is interpreted. Nonetheless, the objective of this study was to investigate fungal genomes isolated from deep, oligotrophic marine sediments. This is the first characterization of fungal isolate genomes sequenced from SPG sediments. Specifically, these genomes were investigated for their metabolic potential with specific focus on carbon, nitrogen, and secondary metabolisms.

### High quality draft genomes

Most evidence of fungi and their role in deep sea sediments has so far been provided by amplicon, metagenomic, and metatranscriptomic data [1–4, 7, 8]. Because very few fungal genomes from the marine subsurface have existed until now, previous data had to be mapped to non-subseafloor fungal reference genomes. Genome reference bias limits the ability to detect novel genes and transcripts, and can make differences observed between genomes uncertain [8]. Therefore, it is important that genomes of fungi isolated from the marine subsurface exist in order to more accurately determine their importance in this environment. Transcriptional profiles of each isolate’s genome would further increase the quality and help decrease the unannotated portions. Nonetheless, these genomes should provide useful information for future studies of fungal genomes from the marine subsurface.

### Transposable elements

It is important that organisms with highly repetitive genomic regions go through a repeat masking step prior to annotation since repeats can cause false positive annotations. Isolate *P*. *pacificagyrus*, which has a larger genome size, contained 5.7% more repeats than *P*. *pacificasedimenti*; however, it has been previously observed that the percentage of repeats can vary within the same species of fungi in general [44]. Furthermore, the abundance of repeats in both genomes fell within the range typically observed in continental and marine fungi (3% to 10%) [45]. Transposable elements (TEs) made up a majority of these repeats which are especially interesting because they are known to play important roles in fungal evolutionary history [44]. A high proportion of TEs were unclassified but this is not uncommon in whole genomic studies of fungi and plants [44, 46–48] and identifying TEs is often difficult due to their mutagenic and degenerative characteristics in fungal and plant genomes [44, 47, 49].

### Secondary metabolite synthesis

In energy limited environments like the SPG sediments, fungi might exist amongst prokaryotes and/or other fungi as a competitor for nutrients which would invoke fungi to produce antimicrobials and other secondary metabolites to fight off competing microorganisms and deal with nutrient stress. These metabolites are synthesized through various combinations of NRPS and PKS pathways. Many genes within both *P*. *pacificasedimenti* and *P*. *pacificagyrus* were found to be involved in NRPS and PKS biosynthesis. The mechanisms of the identified putative antimicrobials varied from disruption of fungal cell wall biosynthesis and integrity [50, 51] to disruption of DNA replication, transcription, and translation [52]. Also identified was a nonribosomal siderophore peptide synthase in each genome which are important in antimicrobial and secondary metabolite synthesis, and subsequent competition with bacteria [53, 54]. Previous studies have found evidence of *Penicillium* secondary metabolites genes [4] and transcripts [7, 14] in marine subsurface sediments. Another study verified that cultured *Penicillium* isolates from Antarctic deep-sea sediments had the ability to produce several different antimicrobial compounds [16]. However, the ecological significance of fungal antimicrobials in this environment has yet to be determined. Our findings, in combination with other studies highlight the biotechnological potential of fungi in marine subsurface sediments and warrants further investigation of this environment, which has gone largely unexplored for antimicrobial products [55]. Future work should focus on testing the potential production of said antimicrobials under a simulated stressed environment.

### Stress and repair responses

Both isolates contained many DNA repair genes and the autophagy initiation complex needed for the formation of the autophagosome, but were missing genes ATG10, ATG16, and ATG21. It may be that the high percentage of uncharacterized proteins herein makes it difficult to assess the true completeness of this pathway, but it also possible these genes were simply lost in these two species [56]. Autophagy is an important conserved pathway amongst fungi that allows them to consume their own necromass (i.e. cytoplasm and organelles) for nutrients under nutrient stress [57–59]. Autophagy has also been found to be a required mechanism before spore formation [60]. A previous metatranscriptomic study from subsurface sediments showed that fungi increased their transcription of both DNA repair and ATG genes in comparison to soil fungi [8]. If metabolically active, it is possible that the fungal isolates herein used DNA repair pathways and autophagocytosis as a means for survival in the oligotrophic sediments of SPG. However, it is important to mention that it is still under debate how much of a role autophagocytosis, i.e. necromass, plays a role in the marine subsurface. Many studies show that amino acid necromass turnover is an important factor for sustaining slow microbial activity [61–64]. But according to Bradley et al. (2018), necromass in general may not provide enough energy to sustain long term microbial populations in SPG sediments without support through the oxidation of buried residual organic carbon from the overlying ocean [65].

Unique to *P*. *pacificagyrus* were genes related to melanin biosynthesis and metal ion transport and response. Melanin is produced in some fungi via the polyketide synthase pathway and largely made for protection against environmental stressors, some of which may be found in subsurface sediments such as, extreme temperatures, heavy metals, and oxidants [66]. Furthermore, it seems that when melanin-containing fungi are under oxidative stress that metal ion response and transport play an important role alongside the pigment to protect the fungi, specifically, melanin binds metal ions thus catalyzing the surrounding free radicals [67–69]. This environmental stress response makes sense when we take into consideration that *P*. *pacificagyrus* was isolated from metalliferous clay in hole U1368D-2H1. Metalliferous clays containing iron-oxide minerals, as in SPG, naturally serve as catalysts for the Fenton-like reactions which produce radicals [70, 71].

### Recalcitrant carbon and nitrogen degradation

In continental and marine environments, fungi are known oxidizers of recalcitrant carbon such as polycyclic aromatic hydrocarbons, lignin, lignocellulose, and carbonate [17, 18, 20, 23, 72] because of their specialized enzymes that can break apart aromatic rings [73]. Due to the similarity of marine subsurface fungi to continental and coastal fungi, it has been proposed that marine subsurface fungi can metabolize recalcitrant carbon in deeply buried marine sediments if they are active [1, 3, 14, 74]. Here, we provide evidence that both isolates have multiple genes coding enzymes which could be used to degrade and consume recalcitrant carbon in SPG sediments.

Much of the recalcitrant carbon in SPG sediments, and marine sediments in general, appears to have been derived from terrestrial input made up of plant matter [70, 75]. Plant cell walls are made up of polysaccharides such as, cellulose, hemicellulose, pectin, and lignin [76, 77]. The double bonded rings in the chemical structure of these polysaccharides makes it inaccessible to most microorganisms [78]. Cytochrome P450 enzymes are not specific to PAH degradation and have been shown to mediate the initial breaking of the rings in lignin [79]. KAAS metabolic analysis and annotations from eggNOG-mapper found that both genomes had several hundred genes related to cytochrome P450 enzymes. Lignolytic enzymes (e.g. laccases, peroxidases, hydrolases, and monooxygenases) are responsible for further degradation of lignocellulosic biomass [78–80]. Evidence of these enzymes were found in both *P*. *pacificagyrus* and *P*. *pacificasedimenti* (Fig. 3) including many genes involved the degradation of double bonded, aromatic compounds found in various recalcitrant carbon sources. Furthermore, we recently confirmed that both isolates could degrade ^13^C labeled lignin both aerobic- and anaerobically [32].

The persistent organic matter composition in the deep sea sediments of SPG largely consists of organic material derived from degraded proteinaceous, nitrogen heavy material, similar to amino acids and peptides [70]. Fungi are unable to fix nitrogen so they must obtain nitrogen needed for growth via assimilatory nitrate reduction and amino acid metabolisms [81]. The amino acids preserved in subsurface sediments could be an accessible nitrogen source for potentially active fungi in these sediments through the use of extra- and intracellular proteases and peptidases that break down this material. Potential fungal expression of these genes was previously reported from 159 mbsf of the Peru Margin [3, 8], providing support for the possibility of fungi to utilize preserved amino acids and peptides in deep sea sediments. We also found that both of the isolates in this study contained the necessary genes to transport amino acids and peptides into the cell, break the compounds down, and use it within biomolecule synthesis pathways (Figs. 3 and 6). These degraded compounds can be used to produce new amino acids and subsequent peptides by converting them to glutamate. At the same time, the residual glutamate is cycled into the TCA cycle, making this a complete extracellular amino acid degradation pathway, where it is used to produce energy for the cell as well.

A byproduct of the TCA cycle is oxalic acid, one of the main organic acids produced by fungi [82]. Oxalic acid can help enhance lignocellulosic biomass degradation as well by balancing the pH surrounding the hyphae which act as a manganese chelator for MnP [83–85]. Another use of oxalic acid is to aid in the degradation of calcium carbonate in order to gain access to important minerals such as iron, manganese, and phosphate that are needed for growth [72]. Evidence of carbonate dissolution by fungi has been observed in subseafloor basalts previously [42, 86, 87], and while this has yet to be reported in SPG sediments, hole U1368D contained 60 wt% to 87 wt% calcium carbonate, making it a possible carbon source for fungi.

## Conclusions

Here, we provide the first constructed genomes of *Penicillium* isolates from deep marine sediments. According to the evidence presented here, we propose *P*. *pacificagyrus* sp. nov. and *P*. *pacificasedimenti* sp. nov. as a new species in the genus *Penicillium*. The ecophysiological characteristics were compared to continental and marine *Penicillium* to further validate our isolates as new species [32]. Both of these novel fungi have the potential to contribute significantly to carbon and nitrogen cycling in the marine subsurface through various degradation pathways, if active. In general, fungi are being overlooked in the marine subsurface environment in regards to nutrient turnover rates. Through these novel genomes, we can understand more confidently what fungi are capable of metabolically in marine subsurface sediments, which has largely been speculated through metagenomic and metatranscriptomic analysis. These genomes provide important information for future studies of marine subsurface fungi.

## Methods

### Sample collection and fungal isolation

The two fungal species in this study were isolated from sediment samples collected during the IODP Expedition 329 to the South Pacific Gyre with the drilling vessel D/V *JOIDES Resolution* [25]. Sites U1368D and U1371E were cored for sediment microbiology (Fig. 1). Potential biological contamination from drilling fluid and seawater intrusion was assessed by the presence of perfluorocarbon microsphere tracers within the interior and exterior of the sediment cores [25]. Fungal isolate *Penicillium* sp. SPG-F1 was isolated from U1371E-14H2 (45°57.8397′S, 163°11.0365′W) at 124 mbsf and *Penicillium* sp. SPG-F15 was isolated from U1368D-2H1 (27°54.9920′S, 123°9.6561′W) at 12 mbsf (Fig. 1). Culture contamination assessment is detailed in Kiel Reese et al. 2021 where both of the isolates were also morphologically and ecophysiologically characterized in conjunction with the whole genome analysis [32].

### Contamination control

Throughout this study, several measures were taken to ensure samples reflected the indigenous microbial community. Samples taken during IODP Expedition 329 were routinely assessed for contamination throughout sample handling through the use of perfluorocarbon tracers, fluorescent microspheres, and visual examination for cracks and other signs of disturbances [25]. Cores for microbiological and molecular purposes were immediately stored in a refrigerated room after collection for subsampling. After sterile subsamples were collected, they were immediately stored at -80ºC. We assessed the purity of our cultured isolates through 18S rRNA clone library sequencing of both the sediments and the isolates. The cultured isolates were identical to the in situ clones from the same samples and depths, which confirmed the cultures were not lab contaminants [32]. Also, all culture transfers and extractions were performed under sterile clean benches while wearing full PPE including face masks and hair bonnets and with sterile reagents which were exposed to UV.

### DNA extraction

High quality genomic DNA (gDNA) was extracted from pure cultures of both *Penicillium* sp. SPG-F1 and *Penicillium* sp. SPG-F15 following a modified cetyltrimethylammonium bromide (CTAB)-based method [88]. Approximately 200 mg of freeze-dried biomass was ground to a powder using a mortar and pestle in the presence of liquid nitrogen. A 500 µl aliquot of extraction buffer (0.25 M NaCl (Nakalai Tesque; Kyoto, Japan), 0.2 M Tris–HCL, pH 8.5 (Nakalai Tesque), 0.025 M EDTA- 2Na (Dojindo Molecular Technologies; Kumamoto, Japan), and 0.5% sodium dodecyl sulfate (FUJIFILM Wako Pure Chemical Corporation; Osaka, Japan)) was added to each extraction. The tubes were vortexed for 10 s and placed in a rotator for 30 min at room temperature. To each extraction, 350 µl of phenol (Nakalai Tesque) was added. The tubes were turned by hand gently 200 times. An aliquot of 150 µl of chloroform (Nakalai Tesque) was added. The tubes were inverted 200 times by hand. The tubes were centrifuged at 20,817 × g for 5 min at ambient temperature. We transferred the supernatant to a new tube containing 700 µl isopropanol (FUJIFILM Wako Pure Chemical Corporation) and centrifuged at 20,817 × g for 5 min at ambient temperature. The DNA was rinsed with 70% ethanol (FUJIFILM Wako Pure Chemical Corporation) and placed in a vacuum evaporator for 10 min. The DNA pellet was resuspended in 240 µl of sterile water and 5 µl of RNase A (10 mg mL^−1^) (Nakalai Tesque). The extractions were incubated at 37 °C for one hour. An aliquot of 40 µl of a 10% CTAB (FUJIFILM Wako Pure Chemical Corporation) and 0.7 M NaCl solution was added to each tube. The extractions were incubated at 67 °C for 10 min. To the tubes, 240 µl of chloroform was added before being centrifuged at 15,294 × g for 5 min. The supernatant was transferred to new tubes filled with 350 µl of phenol and 150 µl of chloroform. The tubes were turned by hand 200 times and centrifuged at 20,817 × g for 5 min. The DNA was precipitated by transferring the supernatant into new tubes containing 2.5 volumes of 100% ethanol and incubating for 20 min at –80 °C. The DNA was centrifuged at 20,817 × g for 5 min. The resulting DNA pellets were rinsed with 70% ethanol and placed into a vacuum evaporator to dry for 10 min. The dried DNA was re-suspended in 50 µl of Tris-buffer (10 mM, pH 8) and placed into a vacuum evaporator to dry before being sent for sequencing.

### DNA sequencing

The DNA extractions for both isolates were quality checked at Macrogen in Kyoto, Japan via a Victor 3 fluorometer and gel electrophoresis prior to library construction. Both genomes were sequenced using an Illumina HiSeq 2000 (Illumina, San Diego, CA) and PacBio RS II (Pacific Biosciences, Menlo Park, USA) sequencer at Macrogen in Kyoto, Japan. An Illumina paired-end library was prepared following the manufacturer’s protocol (TruSeq DNA PCR-Free Sample Preparation Guide, Part # 15,036,187 Rev. A) to generate 101 bp length paired-end reads. The raw reads were quality checked using FastQC (http://www.bioinformatics.babraham.ac.uk/projects/fastqc/). Trim Galore was used to remove the Illumina adapters from the paired-end reads as well as remove low quality reads (quality score [Q] < 20) [89]. For PacBio sequencing, a total of 6 SMRT cells, 3 for each isolate, were sequenced using the DNA Polymerase Binding Kit P6 v2 (Pacific Biosciences, Menlo Park, USA) with an approximate library insert size of 10,000 bp. The three PacBio subreads.fastq files were concatenated into one file for each genome and further processed.

### Genome assembly and annotation

De novo hybrid assembly of the Illumina and PacBio reads was performed using Unicycler [90]. Unicycler first assembled the Illumina reads into contigs and then used the PacBio reads to bridge gaps between the short read contigs. After finalizing the contigs, Unicycler incorporated the program Pilon [91] to polish the assembly by correcting mis-assemblies caused by incorrect bases and gaps. The quality of the assemblies (i.e., the number of contigs, the length of the assembly, the length of the largest contig, and the N50 statistic) was analyzed using QUAST 4.1 [92].

A MAKER2 pipeline was used for annotation without RNA-seq data on small eukaryotic genomes [93]. The pipeline utilizes several ab initio gene prediction programs to iteratively train the gene predictors and produce an accurate gene model. Repetitive elements were identified de novo with RepeatModeler v. 1.0.11 [94]. RepeatMasker was used to mask the repeats in both genomes [95]. RMBlast v. 2.2.28 was used as the search engine. The RepeatMasker edition of RepBase was used as the database [96]. Benchmarking Universal Single-Copy Orthologs (BUSCO) [27] was used to estimate the completeness and contamination of the assembly as well as a training set for the gene predictor Augustus [97, 98]. BUSCO analysis was performed with the fungi_odb10 whereas the training for Augustus was performed with lineage-specific dataset eurotiomycetes_odb9. An unsupervised training gene predictor specific for fungi, GeneMark-ES, was also used [99]. MAKER2 v. 2.31.9 was used to predict protein-coding genes from the masked assembly [100]. Protein evidence was provided using the UniProt protein database [101]. The output from GeneMark-ES and the species folder in Augustus was provided to MAKER2 to enhance the alignment-based gene models. After one iteration of MAKER2, the gene predictions from each contig were combined into one gff3 output file and one zff output file using the accessory scripts *gff3_merge* and *maker2zff*. The zff files were used to train SNAP [102] using the scripts *fathom*, *forge*, and *hmm*-*assembler*.*pl*. The HMM file was incorporated into MAKER2 and gene predictions were ran for a second time. The accessory scripts *fasta_merge* and *gff3_merge* were used to combine the final gene predictions into a gff3 file and a fasta protein file that would be used for phylogenomics and functional gene annotation.

### Phylogenomics

Single-copy homologues from both *Penicillium* isolates were clustered and compared with single-copy homologues from other *Penicillium* and *Aspergillus* genomes downloaded from NCBI and JGI (Additional file 1: Supplementary Table 1) using GET_HOMOLOGUES [36]. The ortholog search was carried out with OrthoMCL using a default cutoff of 1e-05. The genome clusters were compared with GET_HOMOLOGUES script compare_clusters.sh. A maximum-likelihood phylogeny was made with IQ-TREE v. 1.6.1 [37] using 1,000 bootstraps. Best-fit models were chosen according to the Bayesian Information Criterion (BIC) with ModelFinder [38] in IQ-TREE [37]. Tree visualization and tree re-rooting were performed with iTOL [39]. Orthologous gene clusters were further compared between the closest related species to each of our isolates with OrthoVenn2 using an e-value cutoff of 1e-05 and inflation values of 1.5 [40]. AAI’s were estimated using the AAI calculator (http://enve-omics.ce.gatech.edu/).

### Functional gene annotation

Metabolic pathways were determined using the Kyoto Encyclopedia of Genes and Genomes (KEGG) Automatic Annotation Server (KAAS) [30, 31] with fungi set as the genes dataset. The predicted protein-encoding genes were also subjected to CAZy annotation using the DataBase for automated Carbohydrate-active enzyme Annotation (dbCAN) [103], Cluster of Orthologous Groups (COG) annotation using eggNOG-mapper v. 4.5.1 [28, 29], and peptidase classification using BLASTp (e-value 10^–3^) [104] to search against the MEROPS database [105].

## Supplementary Information

**Additional file 1: Supplementary Table 1.** Genome accessions for Fig. 5a. **Supplementary Table 2.** Additional substrate and description information for CAZy annotations in Fig. 3.

## Data Availability

The datasets generated and/or analyzed during the current study are available at NCBI GenBank and NCBI SRA under BioProject IDs PRJNA435890 (https://www.ncbi.nlm.nih.gov/bioproject/PRJNA435890) and PRJNA435885 (https://www.ncbi.nlm.nih.gov/bioproject/PRJNA435885) for *P*. *pacificasedimenti* and *P*. *pacificagyrus*, respectively. Both isolates have been curated at https://www.mycobank.org/ under accessions MB#845416 for *P*. *pacificagyrus* and MB#845451 for *P*. *pacificasedimenti*.
